# Schizophrenia and visual backward masking: a general deficit of target enhancement

**DOI:** 10.3389/fpsyg.2013.00254

**Published:** 2013-05-14

**Authors:** Michael H. Herzog, Maya Roinishvili, Eka Chkonia, Andreas Brand

**Affiliations:** ^1^Laboratory of Psychophysics, Brain Mind Institute, Ecole Polytechnique Fédérale de LausanneLausanne, Switzerland; ^2^Vision Research Laboratory, I.Beritashvili Center of Experimental BiomedecineTbilisi, Georgia; ^3^Institute of Cognitive Neurosciences, Agricultural University of GeorgiaTbilisi, Georgia; ^4^Department of Psychiatry, Tbilisi State Medical UniversityTbilisi, Georgia; ^5^Klinikum Bremen-Ost, Center for Psychiatry and PsychotherapyBremen, Germany

**Keywords:** schizophrenia, vision, acetylcholine receptor, vernier acuity, attention

## Abstract

The obvious symptoms of schizophrenia are of cognitive and psychopathological nature. However, schizophrenia affects also visual processing which becomes particularly evident when stimuli are presented for short durations and are followed by a masking stimulus. Visual deficits are of great interest because they might be related to the genetic variations underlying the disease (endophenotype concept). Visual masking deficits are usually attributed to specific dysfunctions of the visual system such as a hypo- or hyper-active magnocellular system. Here, we propose that visual deficits are a manifestation of a general deficit related to the enhancement of weak neural signals as occurring in all other sorts of information processing. We summarize previous findings with the shine-through masking paradigm where a shortly presented vernier target is followed by a masking grating. The mask deteriorates visual processing of schizophrenic patients by almost an order of magnitude compared to healthy controls. We propose that these deficits are caused by dysfunctions of attention and the cholinergic system leading to weak neural activity corresponding to the vernier. High density electrophysiological recordings (EEG) show that indeed neural activity is strongly reduced in schizophrenic patients which we attribute to the lack of vernier enhancement. When only the masking grating is presented, EEG responses are roughly comparable between patients and control. Our hypothesis is supported by findings relating visual masking to genetic deviants of the nicotinic α7 receptor (CHRNA7).

## Vision and schizophrenia: findings and a framework

The most obvious symptoms of schizophrenia are related to psychopathology (e.g., delusions, hallucinations, negative symptoms, and disorganization syndrome) and cognitive deficits such as working memory and attention. In addition, schizophrenic show strong perceptual deficits. The causes of schizophrenia may be found on various levels including ones which are not directly linked to phenomenology. Here, we propose that visual deficits in schizophrenic patients are not related to visual deficits *per se* but are a manifestation of a general dysfunction of enhancing and stabilizing neural activity.

### Visual backward masking deficits

To understand visual deficits in schizophrenia, we are using a backward masking procedure, called the shine-through effect (Herzog and Koch, 2001; Herzog and Fahle, 2002). This masking technique comes with a two step procedure. First, a vernier stimulus is presented which comprises two vertical bars slightly offset in the horizontal direction. Vernier offset discrimination is a challenging task. Offsets are often smaller than the diameter of a photoreceptor in the retina and, therefore, offset discrimination needs information integration across nearby neurons. Per trial, the vernier is randomly offset either to the left or right. Observers indicate the offset direction. For long vernier durations, schizophrenic patients show no or only very weak deficits. We presented the vernier with different durations starting off with a duration of 150 ms. For this duration, we found thresholds of 41.1 arc sec in patients and 33.0 arc sec in controls. Block by block, we reduced the vernier duration further until observers could not reliably perceive a vernier offset size of 0.66 arc min. This critical vernier duration was 43.2 ms for patients and 27.4 ms for controls (Chkonia et al., 2010). These results are in line with previous studies in which also significant, but moderate, visual deficits were found with different types of visual stimuli (e.g., Saccuzzo et al., 1974; Braff and Saccuzzo, 1981; Saccuzzo and Schubert, 1981).

**Figure 1:**
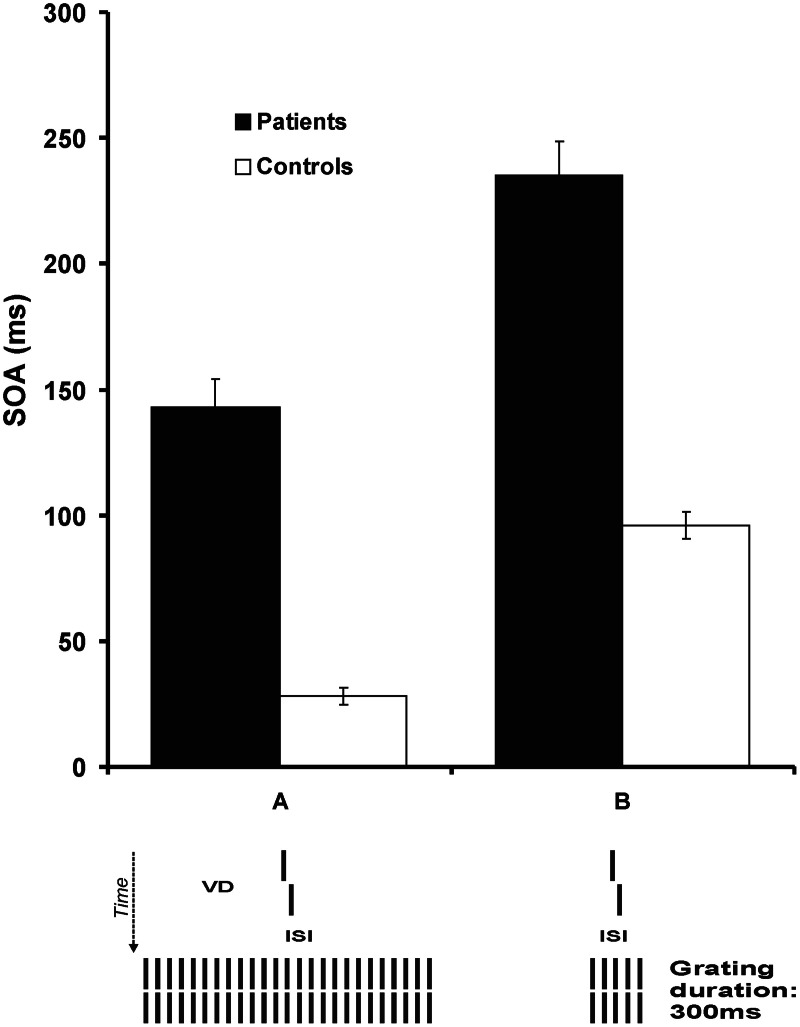
**Step 1.** We presented a vernier without a mask (not shown). In a block of 80 trials, we presented the vernier with a certain duration and determined adaptively the offset size for which 75% correct responses occurred. In successive blocks, we reduced the vernier duration (VD) from 150 ms to the VD for which the vernier offset size was about 0.66′ (arc min). These critical vernier durations were 43.2 ms for patients and 27.4 ms for controls on average. **Step 2.** The vernier was presented with the individual vernier duration of each observer, as determined in step 1. Next a blank screen, i.e., an inter-stimulus-interval (ISI), followed and then a masking grating. The vernier offset was fixed and we adaptively varied the ISI between vernier disappearance and the grating onset. We determined the SOA for which 75% correct responses occurred. We plot results as SOA = VD + ISI. Patients need much longer SOAs than controls. The main effect is due to the prolonged ISIs rather than the only slightly longer vernier durations. Reprinted with permission from Herzog et al. (2004).

To challenge the visual system, we added to the spatially demanding vernier offset discrimination task a temporal challenge by presenting a mask after the vernier. For each observer, we presented the vernier with his/her individual duration as determined in the first step. After the vernier, a blank screen followed, i.e., an inter-stimulus-interval (ISI), and then a mask. In the basic masking conditions, the mask comprised 5 or 25 aligned verniers, i.e., verniers without an offset (Figure 1).

Whereas we varied the vernier offset in the previous condition, here, we kept the vernier offset constant and varied the ISI between the vernier and the mask. We determined the critical ISI for which observers reached 75% correct responses. We found a tremendous deterioration of performance for the schizophrenia patients. With the 25 element grating mask, schizophrenia patients needed SOAs of about 150 ms whereas controls needed only about 30 ms (Herzog et al., 2004; Chkonia et al., 2010). With the 5 element grating mask, patients needed SOAs of about 240 ms while controls needed only about 80 ms (Herzog et al., 2004). Hence, schizophrenic patients show very strong visual deficits when their visual system is challenged both spatially and temporally. These results are well in agreement with previous studies on masking where patients showed strong masking deficits even when target duration was adjusted for each observer individually (e.g., Braff and Saccuzzo, 1981; Green et al., 1994a; Cadenhead et al., 1997).

### Visual backward masking deficits are target enhancement deficits

In the next, third step, we used for each observer his or her individual vernier duration and the individual ISI of the 25 element mask, i.e., we used on average an SOA of 150 ms for the patients and an SOA of 30 ms for the controls. In this condition, we determined the vernier offset size adaptively, i.e., we determined the offset for which 75% correct responses occurred. Because of these normalized, individual values, performance was comparable across all observers for the 25 element grating, i.e., as aimed for.

**Figure 2:**
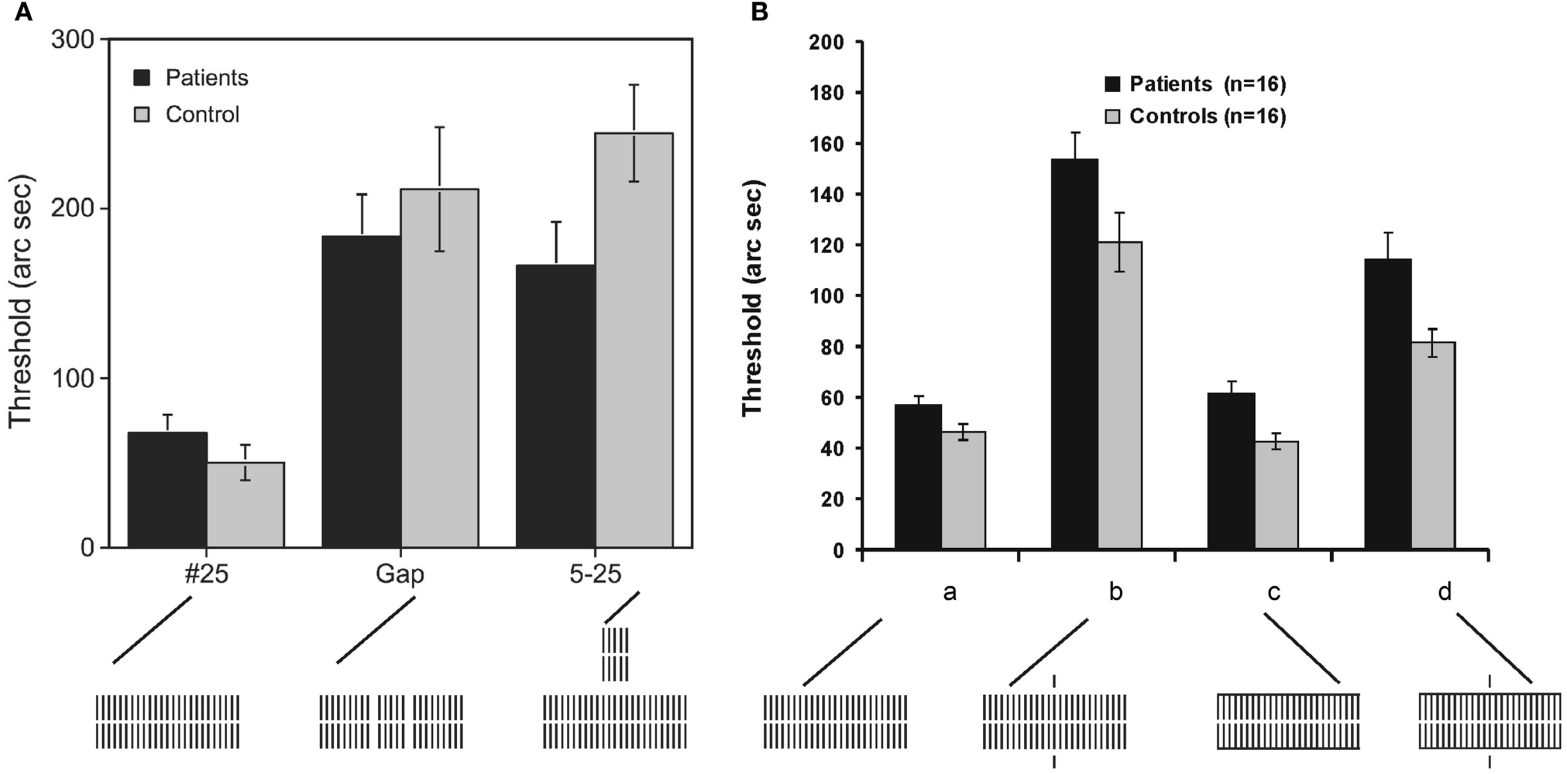
**A vernier was followed by a variety of gratings (the vernier is not shown, only the masking gratings). (A)** Because we used for each observer his/her individual vernier duration and ISI, performance is roughly the same in patients and controls when the 25 element grating follows the vernier. Performance deteriorates significantly for the patients and healthy controls if either a “gap grating” or a “5–25” element grating follows the vernier. In the 5–25 grating, a 5 element grating is presented for 20 ms followed immediately by a 25 element grating for 280 ms. Because of the short duration, the 5 element grating is invisible. Performance deficits are similar in patients and controls indicating, paradoxically, intact spatial and temporal processing of the schizophrenic patients, i.e., patients seem to process the gaps and the briefly presented 5 element grating “carefully.” Were the gaps fully blurred or the 20 ms 5 element grating smeared out, performance would be on the level of the homogeneous 25 element grating (#25). However, this is not the case. **(B)** (a) As in **(A)**, performance of patients and controls is roughly identical with the homogenous 25 element grating because we used the individual vernier durations and ISIs. (b) Performance strongly deteriorates by adding single collinear lines. (c) Horizontal contextual lines yield a performance level comparable to the standard condition (a). (c) Combining vertical and horizontal lines from the conditions (b) and (c) improves performance compared to (b). The horizontal contextual lines “counteract” the vertical lines. Again, it seems that patients “carefully” process the task irrelevant vertical lines, reflected in the strong performance deficits. This deterioration and the recovery from it by horizontal lines, is very similar to the one of controls. Reprinted with permission from Herzog et al. (2004) and Roinishvili et al. (2008).

Next, we removed 2 elements from the 25 element grating creating two gaps, thus, singling out a central grating with 5 elements from two peripheral gratings with 9 elements each (Figure 2). In healthy controls, we showed previously that this gap grating leads to much stronger masking than the homogenous 25 element grating (Herzog and Koch, 2001). We proposed that complex, spatial processing causes the deterioration of performance because nearby elements group together and ungroup from the peripheral elements. Our computer simulations showed how vernier related activity is dynamically suppressed with the gap grating but not with the homogeneous 25 element grating (Herzog et al., 2003).

The rationale of step 3 is as follows. If the gaps, for example, are blurred, performance should, paradoxically, be better than if vision is intact and the gaps are clearly processed. Hence, deterioration of performance with the gap grating indicates intact spatial processing. We found that, indeed, patients showed as strong performance deficits as controls (Figure 2). The very same holds true for temporal manipulations. We presented a 5 element grating for only 20 ms before the 25 element grating lasting for 280 ms. Because of the short duration of 20 ms, the 5 element grating is invisible. Still, it exerts strong performance deficits by a factor of about 5 compared to the 25 element grating. Schizophrenic patients show very similar deteriorations. Hence, it seems that spatial and temporal processing of the masking gratings is largely intact in schizophrenic patients.

In a series of further experiments, we supported these results. For example, we first deteriorated performance by adding small lines to the 25 element grating. Then we added additional horizontal lines which undid the deleterious effects of the vertical lines. Again, performance in patients and controls was rather similar (Schütze et al., 2007; Roinishvili et al., 2008). Hence, it seems that spatio-temporal processing is largely intact in the schizophrenic patients—after vernier duration and SOA were adjusted individually. For this reason, we propose that schizophrenic patients are just two steps from normal (in visual processing). There is a moderate deficit for the unmasked vernier, which could partially reflect long term suffering from the disease. Adolescents with schizophrenia do not show such deficits (Rund et al., 1996; Holzer et al., 2009; see also Saccuzzo et al., 1974). Such deficits may also likely occur when people are suffering, for example, from fever and other diseases affecting attention and concentration. To compensate for these deficits, we provided individually adjusted vernier durations in the masking conditions. Here, we found dramatically deteriorated performance which we attribute to a reduced target enhancement.

### Multifactorial target enhancement

It seems that visual processing of the masks is intact in schizophrenic patients. We propose what is deficient in schizophrenia is the processing of the *target* as a *target*, particularly, in demanding situations when, for example, targets are presented briefly, masked, or their contrast is low (e.g., Slaghuis, 2004). The target is the element of a visual scene which is task relevant. In many everyday situations, there is no target, for example, when observers just passively watch a scene or a movie. In the laboratory, many features or elements can be defined as the target for the very same stimulus. For example, we may have varied the vernier offset direction and, in addition, the length of the grating elements being a bit longer or shorter. In this example, we can ask observers to attend to the vernier offset or to the length of the grating elements. The stimuli are the same, just the tasks differ. Likewise, in a soccer game, you may attend to the actions of the players of team A, or B, or the behavior of other fans.

**Figure 3:**
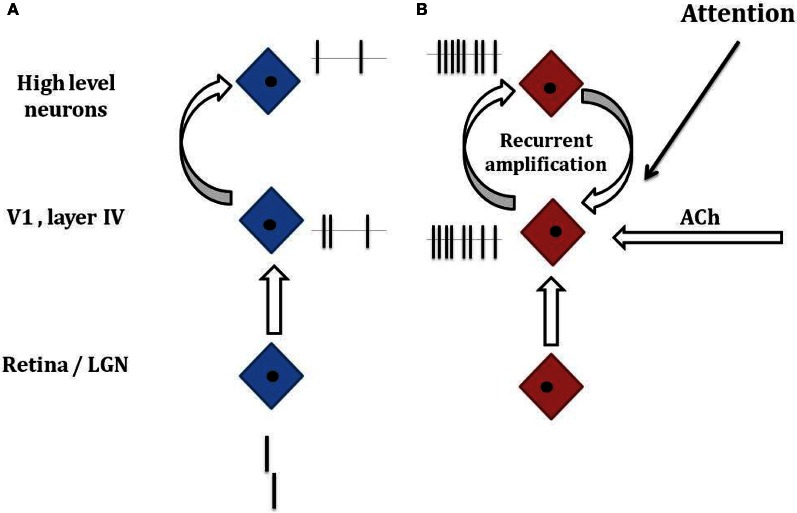
**Hypothetical mechanisms of target enhancement. (A)** A vernier is presented and processed along the visual hierarchy including the retina, the LGN, the primary visual cortex V1, and higher visual areas. Because of the very short presentations times (20–40 ms), only a weak neural response is elicited (small ticks next to the neurons indicated by the blue diamonds). **(B)** If there is target enhancement by recurrent processing, attention, ACh neuromodulation, or other factors, the weak response of the vernier is amplified. Even if the vernier has disappeared on the screen, neural responses may be present because of recurrent amplification. If a masking grating follows the vernier (not shown), recurrent amplification is interrupted and other forms of target enhancement are needed to reach good performance. If target enhancement is dysfunctional, a weaker enhancement leads to poorer performance (as in **A**). These considerations are highly simplified and the underlying mechanisms are largely unknown. Attention is likely to play an important role because without attention to the vernier, performance is strongly deteriorated in healthy observers. Our main proposal is that weak target stimuli need task-dependent enhancements and that these may be dysfunctional in schizophrenic patients.

Vernier offset discrimination is a demanding task when the vernier offset is small and its duration short. In this situation, neural responses are weak and it may be necessary to boost the corresponding weak neural signals to reach good performance. One option is to use recurrent processing (Figure 3). This can lead to strong and persistent neural signals even when the vernier is not presented anymore on the screen. It may be that such recurrent processing is deteriorated in schizophrenia. Because there is no mask, patients can reach the same performance level as controls, after longer recurrent processing. Hence, there are no strong performance deficits. When, however, a mask is presented, the mask may override and interrupt the recurrent vernier processing. To counteract, at least partially, the effects of such masks (and other adverse effects), the human brain is equipped with further mechanisms to enhance weak neural signals.

#### Neuromodulation and attention

We can only speculate on the mechanisms of target enhancement. Next to recurrent processing, one obvious candidate is attention. Attention deficits are core deficits in schizophrenia (e.g., Cornblatt and Keilp, 1994; Nestor and O'Donnell, 1998; Green, 2006). There are many types of attention. One important type is selective visual attention. Visual attention can improve performance substantially. For example, in a classical Posner cueing paradigm, observers fixate a dot in the center of a computer screen. A square appears randomly either on the left or right of the fixation dot. Reaction times are much faster when the cue is presented just before the square at the same location, i.e., when attention can move to the target location before target presentation (Posner and Petersen, 1990). Reaction times are longer when there is no cue or when the cue appears to the “incorrect” side. EEG recordings show higher amplitudes when the target square is correctly cued compared to when not (Hillyard and Anllo-Vento, 1998). Similarly, monkey studies have shown that neural signals can be strongly enhanced when an element in a visual scene is attended compared to when it is not attended (Treue and Martínez-Trujillo, 1999; Maunsell and Cook, 2002). Importantly, only weak, low contrast stimuli benefit from attention (Treue and Maunsell, 1996; Reynolds and Heeger, 2009). We propose that a similar scenario applies to masking situations where target stimuli are weak because of brief presentation times (Figure 3).

Neuromodulation by the cholinergic system might be another mechanism for target enhancement (Cullum et al., 1993; Parasuraman and Greenwood, 2004; Deco and Thiele, 2009). The cholinergic system projects to layer IV of the primary visual cortex where retinal information first enters the visual cortex (Gil et al., 1997; Disney et al., 2007). It is often proposed that acetylcholine controls the influx of “external information” into the cortex. For example, when concentrating on a difficult mathematical problem in a noisy environment, acetylcholine may “shut down” disturbing visual signals, e.g., from a TV screen. In contrast, acetylcholine boosts weak but important information. In psychophysical experiments, targets are presented over and over again in a stereotyped manner and thus target occurrence is predictable, i.e., acetylcholine release can be initiated before target presentation (Sarter et al., 2009). Hence, a hypofunction of the cholinergic system may explain deteriorated vision in schizophrenia (Figure 3).

This proposal is in line with several studies showing that the nicotinic ACh system has significant impact on the mismatch negativity (MMN) (Knott et al., 2012) and sensory gating in schizophrenic patients (Freedman et al., 1997; Adler et al., 1998). In addition, deficits of schizophrenic patients in P50 gating could be improved by nicotine agonists (Adler et al., 2005; Zhang et al., 2012). Hence, a dysfunction of the cholinergic system may be involved in a large variety of dysfunctions including visual ones.

For simple tasks with large and high contrast stimuli, no target enhancement is needed. Accordingly, in these conditions performance is usually quite good in schizophrenic patients. When the verniers are not the target they are likely to go unnoticed. This makes sense because, in this case, vernier enhancement would just lead to amplification of a “disturbing” signal. Hence, target enhancement is activated only when an element is weak and the task-relevant target. Particularly at this stage, deficits of schizophrenia become evident.

However, at present, these considerations remain speculative. There may be more mechanisms and systems that can up- or down-regulate neural activity. In addition, the cholinergic system is a complex system with combinations of several sub-systems (nicotinic vs. muscarinic) which all (or some of them) may be altered in schizophrenia in various combinations. It is likely that not only one enhancing system is deficient in schizophrenia but certain combinations of them.

In addition, very little is known on the effects of acetycholine and attention on vision and how these systems relate to each other and to other systems and mechanisms, including recurrent and top-down neural activation. In addition, it is unclear to which extent acetylcholine is involved in attention itself. Whatever the neural mechanisms are, we propose that there are systems that can enhance targets and that some of them are dysfunctional in schizophrenia.

#### Genetics

Schizophrenic patients are usually heavy smokers (Dickerson et al., 2013) and smoking is often considered a type of self-medication which is very much in accordance with deficits in the cholinergic system (Moran et al., 2012). In schizophrenia, the gene for the alpha 7 receptor subunit was found to be related to deficits in sensory gating (Raux et al., 2002; Houy et al., 2004; Martin et al., 2007). In addition, visual acuity was found to be low in CHRNA7 knockout mice (Origlia et al., 2012). We investigated five single nucleotide polymorphism (SNPs) of CHRNA7 and found that one SNP correlated well with the diagnosis of schizophrenia. Moreover, this SNP showed a high correlation with backward masking deficits in the shine-through effect (Bakanidze et al., 2009).

### EEG

**Figure 4:**
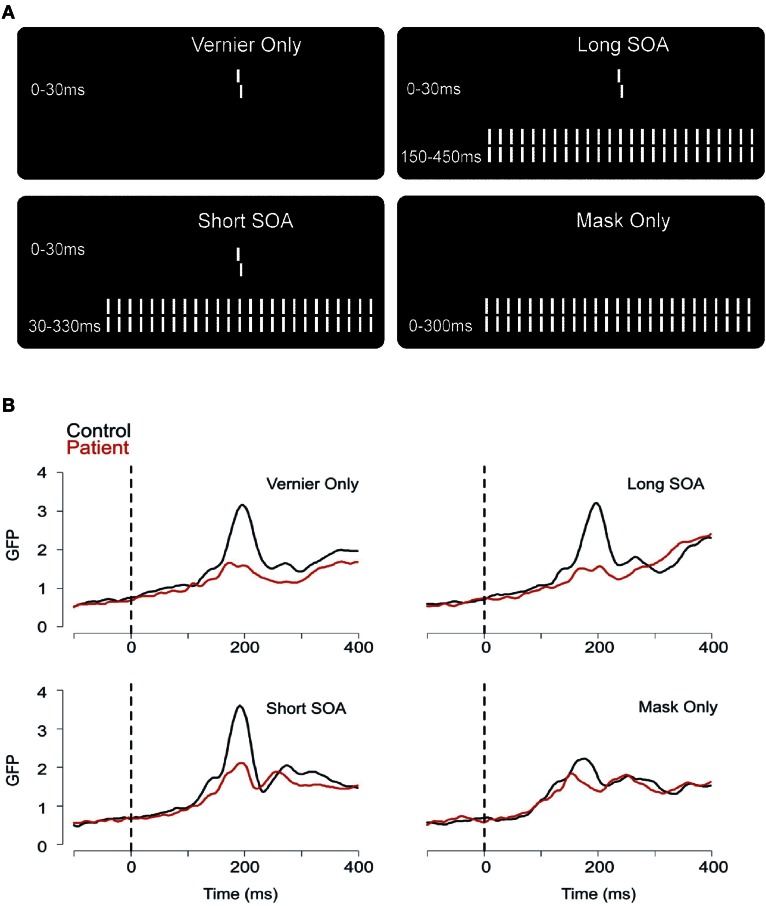
**(A)** In the first condition, a vernier was presented for 30 ms. Observers discriminated the offset direction (here, a right offset is shown). In the second and third conditions, the 30 ms vernier was followed by a grating either immediately (short SOA) or after a blank screen of 120 ms (long SOA). Fourth, we presented only the masking grating. **(B)** The global field power (GFP) for patients and controls in the 4 stimulus conditions showed clear differences in peak amplitude between patients and controls, at around 200 ms [reprinted with permission from Plomp et al. (2013)].

Our model of target enhancement is corroborated by our EEG recordings (Plomp et al., 2013). We recorded brain activity with 64 electrodes and computed the global field power (GFP). The GFP reflects overall brain activity. We determined performance in four conditions (Figure 4A). First, only the vernier was presented. Second, the vernier was followed immediately by the 25 element grating (mean SOA of controls), or, third by an ISI and then by the grating (mean SOA of patients). Fourth, only the grating was presented (no preceding vernier; observers were not aware that there is no vernier because masking is so strong that the vernier is often invisible also when presented). The GFP of patients was much lower than the GFP of controls in the first three conditions where the vernier was presented (Figure 4B). As a speculation, we like to argue that the reduced GFP reflects a lack of target enhancement of the vernier. The reduced neural activity becomes behaviorally relevant only in the two masked conditions because, as we argue, recurrent processing is disturbed by the mask and, thus, target amplification is reduced. In the vernier only condition, longer recurrent processing may compensate the reduced neural activity (see Neuromodulation and Attention). Reduced EEG traces may also be caused by other factors such as diminished neural excitation. However, interestingly, EEG traces of patients and controls were very similar in the mask only condition arguing against a permanent deficit (Figure 4B).

## Discussion

We view visual masking deficits in schizophrenia as a manifestation of a general deficit of target enhancement rather than as a specific visual deficit such as a dysfunction of the magnocellular system (see below). In this sense, visual dysfunctions in schizophrenia allow a *general* view into the basic “mechanisms of madness.” We suggest that mechanisms similar to target enhancement may by deficient also in other visual and cognitive processes of schizophrenic patients.

For example, schizophrenic patients show strong deficits in the detection of low contrast stimuli (Slaghuis, 1998, 2004; Kéri et al., 2004; Calderone et al., 2013). It remains an open question whether these visual deficits are restricted to patients with negative symptoms (e.g., Slaghuis, 1998) and to certain stimuli, such as low spatial frequency gratings (Kéri et al., 2004). Delord et al. (2006) found that contrast detection thresholds of schizophrenic patients were significantly poorer than those of controls for all frequencies, arguing rather for a general visual deficit. Patients show also strongly deteriorated performance for high contrast stimuli when these stimuli are fragmented, i.e., where only parts of the contour of an object are visible. We like to argue that all these tasks require strong attention and some sort of task stabilization because information has to be integrated, for example, across contour elements in a time consuming manner.

In a similar fashion, we think that target enhancement is crucial in cognition. For the basic continuous performance test (CPT), performance is only slightly deteriorated in patients compared to controls (Nuechterlein et al., 1992). However, when stimuli are embedded in noise and, thus, contrast reduces, performance of patients deteriorates much more strongly than performance in controls. Effects are more pronounced when a memory component is added (Nuechterlein et al., 1992; Chkonia et al., 2010). We like to argue that these tasks, as the masking paradigm, require mechanisms to stabilize information across space and time, and that this ability is deteriorated in schizophrenic patients. The cholinergic system makes projections throughout the entire brain and can thus stabilize all sorts of processing including the ones mentioned above (Nelson et al., 2005; Deco and Thiele, 2009; Bentley et al., 2011; Yakel, 2013). The same holds true for attention.

Schizophrenic patients show also deficits in passive tests such as the MMN (Olincy and Freedman, 2012), prepulse inhibition (PPI), and P50 gating (Adler et al., 1991; Olincy et al., 2010). We will argue below that we do not propose that dysfunctional target enhancement is the only deficit in schizophrenic patients. To the contrary, we suggest that there are many deficits necessary to create the full blown symptoms of schizophrenia. Hence, MMN and P50 gating deficits may be of a different nature than masking deficits. In this sense, it is surprising that nicotinic agonists can compensate P50 deficits (Adler et al., 2005; Zhang et al., 2012) and MMN deficits (Knott et al., 2012). In addition, we like to mention that schizophrenic patients show also immune deficits such as a diminished niacin skin response (Puri et al., 2001; Messamore, 2012). Also these deficits can, surprisingly, be linked to the cholinergic system (Gallowitsch-Puerta and Tracey, 2005). Hence, cholinergic deficits may be much more fundamental and not be related to target enhancement only.

Schizophrenic patients show often a strong degree of cognitive disorganization affecting performance in a large variety of everyday tasks. For this reason, cognitive disorganization is, next to positive and negative symptoms a key aspect in the psychopathology of schizophrenia. One century ago, Bleuler proposed that loose associations are even the basic symptoms of schizophrenia and can cause delusions and hallucinations (Bleuler, 1911). We found that persons with high scores of cognitive disorganization of schizotypy have problems focusing on tasks which may be related to stabilizing task related information. Accordingly, we found backward masking deficits in the shine-through paradigm in healthy psychology students with high scores on cognitive disorganization but not with high scores for the positive and negative dimensions (Cappe et al., 2012).

We do not propose that attention and cholinergic dysfunctions are necessary and sufficient for schizophrenia. Likewise, we do not propose that target enhancement is necessary and sufficient for schizophrenia. Quite to the contrary, we believe that dysfunctional target enhancement is only one factor that can contribute to schizophrenia. First, there are patients without target enhancement deficits since not all patients have masking deficits. On the other hand, healthy student observers can have (moderate) masking deficits without an indication of the disease. Second, there can be many causes for dysfunctional neural enhancements. Attention and cholinergic modulation are just two examples. Within the cholinergic system, there may be many deficits leading to a similar phenotype since the cholinergic system itself is a complex system with two subsystems and various receptor types. In addition, there may be many mutations that all can lead to one dysfunction. For this reasons, we suggest that the study of endophenoytpes can be a successful tool to characterize subpopulations of schizophrenia. Visual masking is only one potential endophenotype.

### Endophenotype concept

The shine-through masking paradigm is a potential endophenotype of schizophrenia. We found masking deficits not only in schizophrenic patients but also in their unaffected relatives (Chkonia et al., 2010). In addition, masking deficits were stable for 1 year (Chkonia et al., 2010) and adolescents with psychosis show masking deficits even before the manifestation of the disease (Holzer et al., 2009). Masking deficits are specific for the spectrum of functional psychoses (bipolar, schizoaffective, and schizophrenic patients) and were not found in depressed patients and abstinent alcoholics (Chkonia et al., 2012). We like to mention that visual backward masking can easily be controlled and has a much better signal to noise ratio than most cognitive tests (Chkonia et al., 2012).

### The dysfunction hypothesis of the magnocellular system

Almost all current approaches on visual masking deficits in schizophrenia propose a dysfunction of the magno-cellular system based on the dual channel model of Breitmeyer and Ganz (1976). Enhanced masking in schizophrenic patients is attributed to either a hyperactive or hypoactive magno-cellular visual system (e.g., Green et al., 1994a,b; Schechter et al., 2003; Slaghuis, 2004; Butler et al., 2007). A full review is beyond the scope and goal of this article [for critical reviews, see Skottun and Skoyles (2007, 2009)]. We just like to mention that most masking research in schizophrenic patients investigated A-type masking whereas the dual channel model is mainly proposed to explain B-type masking (non-monotonic masking functions with strongest masking for SOAs of around 50 ms). Also the shine-through paradigm reveals A-type masking characteristics and stimuli are biased rather toward the parvo-cellular than the magnocellular system.

Our proposal shares many similarities with early models on visual masking deficits in schizophrenia where a key component is target storage in an iconic memory. The iconic memory protects target related activity to be overwritten by the mask. Masking deficits in schizophrenia patients were proposed to occur by a dysfunctional storage mechanism or slow information transfer to short term memory (Saccuzzo et al., 1974; Braff, 1981; Saccuzzo and Braff, 1981; Schwartz et al., 1983; for a review see Schuck and Lee, 1989).

## Summary

We found strong deficits with the shine-through masking paradigm in schizophrenic patients (Herzog et al., 2004), in accordance with many other studies on visual masking (Saccuzzo et al., 1974; Braff, 1981; Saccuzzo and Braff, 1981; Green et al., 1994a,b). Also patients with other functional psychoses than schizophrenia show strong masking deficits; however, there are no deficits in unipolar depressive patients (Chkonia et al., 2012). The shine-through masking paradigm is a potential endophenotype of schizophrenia because, amongst other findings, relatives of schizophrenic patients show masking deficits (Chkonia et al., 2010), masking deficits occur before the onset of schizophrenia in adolescents with psychosis (Holzer et al., 2009), and in healthy students with high schizotypy scores (Cappe et al., 2012). On the neural level, we found reduced EEG traces (Plomp et al., 2013) and, on the genetic level, an abnormal SNPs related to the cholinergic system (Bakanidze et al., 2009).

We propose the following speculative model which closes the loop linking phenomenological findings (masking deficits) to genetic abnormalities which, in turn, cause deviant neural processing as evident in the EEG. First, visual processing *per se* is largely intact in schizophrenic patients as evidenced in good performance in visual acuity tests and the vernier task with long vernier durations. Even for short vernier durations, only mild deficits occur. We related these deteriorations partly to unspecific effects as they may occur from suffering from a chronic disease including severe medication effects. Second, there are pronounced masking deficits which we relate to specific deteriorations of target enhancement and stabilization. Third, we propose that a deficient cholinergic system is one out of possibly more deficient enhancement mechanisms. Heavy smoking of patients may be an attempt to compensate for the deficient cholinergic system. Fourth, reduced GFP in the EEG may be an indication of deficient target enhancement. Fifth, we view masking deficits as a manifestation of similar enhancement deficits as they may occur in cognition, emotion, and personality. Sixth, we do *not* propose that deficient target enhancement is necessary and sufficient for schizophrenia. To the contrary, there are schizophrenic patients without masking deficits. On the other hand, enhancement deficits are not sufficient for schizophrenia because there are healthy students with masking deficits. In addition, there are potentially many systems that contribute to information enhancement and likely a combination of these mechanisms has to be deviant to cause schizophrenia.

### Conflict of interest statement

The authors declare that the research was conducted in the absence of any commercial or financial relationships that could be construed as a potential conflict of interest.

## References

[B1] Adler L. E. Cawthra E. M. Donovan K. A. Harris J. G. Nagamoto H. T. Olincy A. (2005). Improved p50 auditory gating with ondansetron in medicated schizophrenia patients. Am. J. Psychiatry 162, 386–388 10.1176/appi.ajp.162.2.386 15677607

[B2] Adler L. E. Olincy A. Waldo M. Harris J. G. Griffith J. Stevens K. (1998). Schizophrenia, sensory gating, and nicotinic receptors. Schizophr. Bull. 24, 189–202 9613620 10.1093/oxfordjournals.schbul.a033320

[B2a] Adler L. E. Waldo M. C. Tatcher A. Cawthra E. Baker N. Freedman R. (1991). Lack of relationship of auditory gating defects to negative symptoms in schizophrenia. Schizophr. Res. 3, 131–138 2278977 10.1016/0920-9964(90)90046-a

[B3] Bakanidze G. Puls I. Brand A. Herzog M. H. Roinishvili M. Chkonia E. (2009). The influence of genetic variants of the CHRNA7 gene on backward masking in schizophrenic patients. Eur. Arch. Psychiatry. Clin. Neurosci. 259 Suppl. 1, S101

[B4] Bentley P. Driver J. Dolan R. J. (2011). Cholinergic modulation of cognition: insights from human pharmacological functional neuroimaging. Prog. Neurobiol. 94, 360–388 10.1016/j.pneurobio.2011.06.002 21708219 PMC3382716

[B5] Bleuler E. (1911). Dementia praecox oder Gruppe der Schizophrenien. Leipzig; Wien: F. Deuticke

[B6] Braff D. L. (1981). Impaired speed of information processing in nonmedicated schizotypal patients. Schizophr. Bull. 7, 499–508 10.1093/schbul/7.3.499 7280576

[B7] Braff D. L. Saccuzzo D. P. (1981). Information processing dysfunction in paranoid schizophrenia: a two-factor deficit. Am. J. Psychiatry 138, 1051–1056 7258380 10.1176/ajp.138.8.1051

[B8] Breitmeyer B. G. Ganz L. (1976). Implications of sustained and transient channels for theories of visual pattern masking, saccadic suppression, and information processing. Psychol. Rev. 83, 1–36 766038

[B9] Butler P. D. Martinez A. Foxe J. J. Kim D. Zemon V. Silipo G. (2007). Subcortical visual dysfunction in schizophrenia drives secondary cortical impairments. Brain 130, 417–430 10.1093/brain/awl233 16984902 PMC2072909

[B10] Cadenhead K. S. Geyer M. A. Butler R. W. Perry W. Sprock J. Bradd D. L. (1997). Information processing deficits of schizophrenia patients: relationship to clinical ratings, gender and medication status. Schizophr. Res. 28, 51–62 9428064 10.1016/s0920-9964(97)00085-6

[B11] Calderone D. J. Martinez A. Zemon V. Hoptman M. J. Hu G. Watkins J. E. (2013). Comparison of psychophysical, electrophysiological, and fMRI assessment of visual contrast responses in patients with schizophrenia. Neuroimage 67, 153–162 10.1016/j.neuroimage.2012.11.019 23194815 PMC3544989

[B12] Cappe C. Herzog M. H. Herzig D. A. Brand A. Mohr C. (2012). Cognitive disorganisation in schizotypy is associated with deterioration in visual backward masking. Psychiatry Res. 200, 652–659 10.1016/j.psychres.2012.07.001 22921599

[B13] Chkonia E. Roinishvili M. Makhatadze N. Tsverava L. Stroux A. Neumann K. (2010). The shine-through masking paradigm is a potential endophenotype of schizophrenia. PLoS ONE 5:e14268 10.1371/journal.pone.0014268 21151559 PMC3000331

[B14] Chkonia E. Roinishvili M. Reichard L. Wurch W. Puhlmann H. Grimsen C. (2012). Patients with functional psychoses show similar visual backward masking deficits. Psychiatry Res. 198, 235–240 10.1016/j.psychres.2012.02.020 22464992

[B15] Cornblatt B. A. Keilp J. G. (1994). Impaired attention, genetics, and the pathophysiology of schizophrenia. Schizophr. Bull. 20, 31–46 10.1093/schbul/20.1.31 8197420

[B16] Cullum C. M. Harris J. G. Waldo M. C. Smernoff E. Madison A. Nagamoto H. T. (1993). Neurophysiological and neuropsychological evidence for attentional dysfunction in schizophrenia. Schizophr. Res. 10, 131–141 8398945 10.1016/0920-9964(93)90048-n

[B17] Deco G. Thiele A. (2009). Attention: oscillations and neuropharmacology. Eur. J. Neurosci. 30, 347–354 10.1111/j.1460-9568.2009.06833.x 19614749 PMC2777251

[B18a] Delord S. Ducato M. G. Pins D. Devinck F. Thomas P. Boucart M. (2006). Psychophysical assessment of magno- and parvocellular function in schizophrenia. Vis. Neurosci. 23, 645–650 10.1017/S0952523806233017 16962008

[B19] Dickerson F. Stallings C. R. Origoni A. E. Vaughan C. Khushalani S. Schroeder J. (2013). Cigarette smoking among persons with schizophrenia or bipolar disorder in routine clinical settings, 1999–2011. Psychiatr. Serv. 64, 44–50 10.1176/appi.ps.201200143 23280457

[B20] Disney A. A. Aoki C. Hawken M. J. (2007). Gain modulation by nicotine in macaque V1. Neuron 56, 701–713 10.1016/j.neuron.2007.09.034 18031686 PMC2875676

[B21] Freedman R. Coon H. Myles-Worsley M. Orr-Urteger A. Olincy A. Davis A (1997). Linkages of a neurophysiological deficit in schizophrenia to chromosome 15 locus. Proc. Natl. Acad. Sci. U.S.A. 94, 587–592 9012828 10.1073/pnas.94.2.587 PMC19557

[B22] Gallowitsch-Puerta M. Tracey K. J. (2005). Immunologic role of the cholinergic anti-inflammatory pathway and the nicotinic acetylcholine α7 receptor. Ann. N.Y. Acad. Sci. 1062, 1–11 10.1196/annals.1358.024 16461803

[B23] Gil Z. Connors B. W. Amitai Y. (1997). Differential regulation of neocortical synapses by neuromodulators and activity. Neuron 19, 679–686 10.1016/S0896-6273(00)80380-3 9331357

[B24] Green M. F. (2006). Cognitive impairment and functional outcome in schizophrenia and bipolar disorder. J. Clin. Psychiatry 67, 3–8 discussion: 36–42. 16965182

[B25] Green M. F. Nuechterlein K. H. Mintz J. (1994a). Backward masking in schizophrenia and mania. I. Specifying a mechanism. Arch. Gen. Psychiatry 51, 939–944 10.1001/archpsyc.1994.03950120011003 7979881

[B26] Green M. F. Nuechterlein K. H. Mintz J. (1994b). Backward masking in schizophrenia and mania. II. Specifying the visual channels. Arch. Gen. Psychiatry 51, 945–951 10.1001/archpsyc.1994.03950120017004 7979882

[B28] Herzog M. H. Ernst U. Etzold A. Eurich C. (2003). Local interactions in neural networks explain global effects in Gestalt processing and masking. Neural Comput. 15, 2091–2113 10.1162/089976603322297304 12959667

[B29] Herzog M. H. Fahle M. (2002). Effects of grouping in contextual modulation. Nature 415, 433–436 10.1038/415433a 11807555

[B30] Herzog M. H. Koch C. (2001). Seeing properties of an invisible object: feature inheritance and shine-through. Proc. Natl. Acad. Sci. U.S.A. 98, 4271–4275 10.1073/pnas.071047498 11274451 PMC31215

[B31] Herzog M. H. Kopmann S. Brand A. (2004). Intact figure-ground segmentation in schizophrenia. Psychiatry Res. 129, 55–63 10.1016/j.psychres.2004.06.008 15572185

[B32] Hillyard S. A. Anllo-Vento L. (1998). Event-related brain potentials in the study of visual selective attention. Proc. Natl. Acad. Sci. U.S.A. 95, 781–787 9448241 10.1073/pnas.95.3.781 PMC33798

[B33] Holzer L. Jaugey L. Chinet L. Herzog M. H. (2009). Deteriorated visual backward masking in the shine-through effect in adolescents with psychosis. J. Clin. Exp. Neuropsychol. 31, 641–647 10.1080/13803390802438454 19031325

[B34] Houy E. Raux G. Thibaut F. Belmont A. Demily C. Allio G. (2004). The promoter -194 C polymorphism of the nicotinic alpha 7 receptor gene has a protective effect against the P50 sensory gating deficit. Mol. Psychiatry 9, 320–322 10.1038/sj.mp.4001443 14569275

[B35] Kéri S. Kelemen O. Benedek G. Janka Z. (2004). Vernier threshold in patients with schizophrenia and in their unaffected siblings. Neuropsychology 18, 537–542 10.1037/0894-4105.18.3.537 15291731

[B36] Knott V. Shah D. Millar A. McIntosh J. Fisher D. Blais C. (2012). Nicotine, auditory sensory memory, and sustained attention in a human ketamine model of schizophrenia: moderating influence of a hallucinatory trait. Front. Pharmacol. 3:172 10.3389/fphar.2012.00172 23060793 PMC3460347

[B37] Martin L. F. Leonard S. Hall M. H. Tregellas J. R. Freedman R. Olincy A. (2007). Sensory gating and alpha-7 nicotinic receptor gene allelic variants in schizoaffective disorder, bipolar type. Am. J. Med. Genet. B Neuropsychiatr. Genet. 144B, 611–614 10.1002/ajmg.b.30470 17192894 PMC3123155

[B38] Maunsell J. H. R. Cook E. P. (2002). The role of attention in visual processing. Philos. Trans. R. Soc. Lond. B Biol. Sci. 357, 1063–1072 10.1098/rstb.2002.1107 12217174 PMC1693016

[B39] Messamore E. (2012). Niacin subsensitivity is associated with functional impairment in schizophrenia. Schizophr. Res. 137, 180–184 10.1016/j.schres.2012.03.001 22445461

[B39a] Moran L. V. Sampath H. Kochunov P. Hong L. E. (2012). Brain circuits that link schizophrenia to high risk of cigarette smoking. Schizophr. Bull. [Epub ahead of print]. 10.1093/schbul/sbs149 23236076 PMC3796082

[B40] Nelson C. L. Sarter M. Bruno J. P. (2005). Prefrontal cortical modulation of acetylcholine release in posterior parietal cortex. Neuroscience 132, 347–359 10.1016/j.neuroscience.2004.12.007 15802188

[B41] Nestor P. G. O'Donnell B. F. (1998). The mind adrift: attentional dysregulation in schizophrenia, in The Attentive Brain, ed Parasuraman R. (Cambridge, MA: MIT Press), 527–546

[B41a] Nuechterlein K. H. Dawson M. E. Gitlin M. Ventura J. Goldstein M. J. Snyder K. S. (1992). Developmental processes in schizophrenic disorders: longitudinal studies of vulnerability and stress. Schizophr. Bull. 18, 387–425 10.1093/schbul/18.3.387 1411329

[B42] Olincy A. Braff D. L. Adler L. E. Cadenhead K. S. Calkins M. E. Dobie D. J. (2010). Inhibition of the P50 cerebral evoked response to repeated auditory stimuli: results from the Consortium on Genetics of Schizophrenia. Schizophr. Res. 119, 175–182 10.1016/j.schres.2010.03.004 20382002 PMC3688282

[B43] Olincy A. Freedman R. (2012). Nicotinic mechanisms in the treatment of psychotic disorders: a focus on the α7 nicotinic receptor. Handb. Exp. Pharmacol. 213, 211–232 10.1007/978-3-642-25758-2_8 23027417 PMC3692393

[B44] Origlia N. Valenzano D. R. Moretti M. Gotti C. Domenici L. (2012). Visual acuity is reduced in lpha 7 nicotinic receptor knockout mice. Invest. Ophthalmol. Vis. Sci. 53, 1211–1218 22281823 10.1167/iovs.11-8007

[B45] Parasuraman R. Greenwood P. (2004). Molecular genetics of visuospatial attention and working memory, in Cognitive Neuroscience of Attention, ed Posner M. I. (New York; London: Guilford Press), 245–259

[B46] Plomp G. Roinishvili M. Chkonia E. Kapanadze G. Kereselidze M. Brand A. (2013). Electrophysiological evidence for ventral stream deficits in schizophrenia patients. Schizophr. Bull. 39, 547–554 10.1093/schbul/sbr175 22258884 PMC3627769

[B47] Posner M. I. Petersen S. E. (1990). The attention system of the human brain. Annu. Rev. Neurosci. 13, 25–42 10.1146/annurev.ne.13.030190.000325 2183676

[B48] Puri B. K. Easton T. Das I. Kidane L. Richardson A. J. (2001). The niacin skin flush test in schizophrenia: a replication study. Int. J. Clin. Pract. 55, 368–370 11501224

[B49] Raux G. Bonnet-Brilhault F. Louchart S. Houy E. Gantier R. Levillain D. (2002). The -2 bp deletion in exon 6 of the ‘alpha 7-like’ nicotinic receptor subunit gene is a risk factor for the P50 sensory gating deficit. Mol. Psychiatry 7, 1006–1011 10.1038/sj.mp.4001140 12399955

[B50] Reynolds J. H. Heeger D. J. (2009). The normalization model of attention. Neuron 61, 68–185 10.1016/j.neuron.2009.01.002 19186161 PMC2752446

[B51] Roinishvili M. Chkonia E. Brand A. Herzog M. H. (2008). Contextual suppression and protection in schizophrenic patients. Eur. Arch. Psychiatry Clin. Neurosci. 258, 210–216 10.1007/s00406-007-0780-9 18297426

[B51a] Rund B. R. Øie M. Sundet K. (1996). Backward-masking deficit in adolescents with schizophrenic disorders or attention deficit hyperactivity disorder. Am. J. Psychiatry 153, 1154–1157 8780418 10.1176/ajp.153.9.1154

[B52] Saccuzzo D. P. Braff D. L. (1981). Early information processing deficit in schizophrenia. New findings using schizophrenic subgroups and manic control subjects. Arch. Gen. Psychiatry 38, 175–179 10.1001/archpsyc.1981.01780270061008 7212944

[B53] Saccuzzo D. P. Hirt M. Spencer T. J. (1974). Backward masking as a measure of attention in schizophrenia. J. Abnorm. Psychol. 83, 512–522 4455714 10.1037/h0037072

[B54] Saccuzzo D. P. Schubert D. L. (1981). Backward masking as a measure of slow processing in schizophrenia spectrum disorders. J. Abnorm. Psychol. 90, 305–312 7264060 10.1037//0021-843x.90.4.305

[B55] Sarter M. Lustig C. Taylor S. F. (2012). Cholinergic contributions to the cognitive symptoms of schizophrenia and the viability of cholinergic treatments. Neuropharmacology 62, 1544–1553 10.1016/j.neuropharm.2010.12.001 21156184 PMC3920544

[B56] Sarter M. Parikh V. Howe W. M. (2009). Phasic acetylcholine release and the volume transmission hypothesis: time to move on. Nat. Rev. Neurosci. 10, 383–390 10.1038/nrn2635 19377503 PMC2699581

[B57] Schechter I. Butler P. D. Silipo G. Zemon V. Javitt D. C. (2003). Magnocellular and parvocellular contributions to backward masking dysfunction in schizophrenia. Schizophr. Res. 64, 91–101 10.1016/S0920-9964(03)00008-2 14613674

[B58] Schuck J. R. Lee R. G. (1989). Backward masking, information processing, and schizophrenia. Schizophr. Bull. 15, 491–500 10.1093/schbul/15.3.491 2683042

[B59] Schütze C. Bongard I. Marbach S. Brand A. Herzog M. H. (2007). Collinear contextual suppression in schizophrenic patients. Psychiatry Res. 150, 237–243 10.1016/j.psychres.2006.03.021 17321597

[B60] Schwartz B. D. Winstead D. K. Adinoff B. (1983). Temporal integration deficit in visual information processing by chronic schizophrenics. Biol. Psychiatry 18, 1311–1320 6652164

[B61] Skottun B. C. Skoyles J. R. (2007). Contrast sensitivity and magnocellular functioning in schizophrenia. Vision Res. 47, 2923–2933 10.1016/j.visres.2007.07.016 17825350

[B62] Skottun B. C. Skoyles J. R. (2009). Are masking abnormalities in schizophrenia limited to backward masking? Int. J. Neurosci. 119, 88–104 10.1080/00207450802480168 19116834

[B63] Slaghuis W. L. (1998). Contrast sensitivity for stationary and drifting spatial frequency ggratings in positive- and negative-symptom schizophrenia. J. Abnorm. Psychol. 107, 49–62 10.1037/0021-843X.107.1.49 9505038

[B64] Slaghuis W. L. (2004). Spatio-temporal luminance contrast sensitivity and visual backward masking in schizophrenia. Exp. Brain Res. 156, 196–211 10.1007/s00221-003-1771-3 14752582

[B66] Treue S. Martínez-Trujillo J. C. (1999). Feature-based attention influences motion processing gain in macaque visual cortex. Nature 399, 575–579 10.1038/21176 10376597

[B67] Treue S. Maunsell J. H. (1996). Attentional modulation of visual motion processing in cortical areas MT and MST. Nature 382, 539–541 10.1038/382539a0 8700227

[B67a] Yakel J. L. (2013). Cholinergic receptors: functional role of nicotinic ACh receptors in brain circuits and disease. Pflugers Arch. 465, 441–450 10.1007/s00424-012-1200-1 23307081 PMC3633680

[B69] Zhang X. Y. Liu L. Liu S. Hong X. Chen da C. Xiu M. H. (2012). Short-term tropisetron treatment and cognitive and p50 auditory gating deficits in schizophrenia. Am. J. Psychiatry 169, 974–981 10.1176/appi.ajp.2012.11081289 22952075

